# The Mechanism and Research Progress of Skin Microbiota in Pathogenesis of Acne

**DOI:** 10.1155/drp/9910076

**Published:** 2025-10-14

**Authors:** Xinwei Li, Juan Jin

**Affiliations:** Zhejiang Provincial Engineering Research Center of New Technologies and Applications for Targeted Therapy of Major Diseases, College of Life Science and Medicine, Zhejiang Sci-Tech University, Hangzhou, China

**Keywords:** acne, gut–skin axis, inflammatory skin disease, skin microbiota

## Abstract

Acne is a chronic inflammatory skin disease of the sebaceous unit of the facial hair follicle that occurs mainly in adolescence. The four major pathogenesis of acne are excessive secretion of sebum by sebaceous glands, abnormal keratosis of sebaceous glands in hair follicles, reproduction of skin microorganisms such as Cutibacterium acnes (*C. acnes*), and inflammatory reaction. Among the skin microbiota, *C. acnes* and *Malassezia* affect the secretion of sebaceous glands, mediate inflammation, and are closely related to the pathogenesis of acne. With the development of the theory of “Gut–skin axis,” the role of intestinal microbiota and skin microecology in acne regulation has gradually become the focus of researchers. The purpose of this study is to investigate the influence of skin microbiota and the interaction between gut and skin on the pathogenesis of acne and to analyze the potential mechanism of skin microbiota during the pathogenesis of acne. It is expected that further understanding of skin microbiota (including its potential mechanism) will help clarify its role in acne and provide new ideas for the pathogenesis and clinical treatment of acne and other inflammatory skin diseases.

## 1. Introduction

Skin, as the largest organ of the human body and one of the four major bacterial banks, is colonized by a variety of microorganisms on the skin surface, including bacteria, viruses, fungi, and mites, called skin microbiota, which together with the host skin and the microenvironment on the skin surface constitute a complex ecosystem [1, 2]. The skin microbiota regulates the immune system, participates in metabolic processes, and acts as a biological barrier against external pathogens [1, 3]. Acne is a common chronic inflammatory skin disease occurring in the sebaceous glands of hair follicles, mainly in the area of strong sebum secretion, mostly in the face, and is easy to occur repeatedly, bringing adverse effects on the physical and mental health of patients. According to the shape of skin lesions, acne can be divided into acne type, papular type, pustular type, nodular type, and cyst type, with an incidence of 80% in the adolescent group. Therefore, it is particularly important to understand the formation factors of acne and the corresponding treatment options. Acne is a common chronic inflammatory skin disorder that occurs in the pilosebaceous units. It mainly emerges in areas with vigorous sebum secretion, often on the face, and is highly prone to recurrence, exerting adverse effects on the physical and mental health of patients [4]. Based on the morphology of skin lesions, acne can be classified into comedonal, papular, pustular, nodular, and cystic types, with the incidence rate reaching 80% among the adolescent population. Hence, understanding the formation factors of acne and the corresponding therapeutic regimens is of particular significance.

Acne is a multifactorial skin disease, whose onset is mainly related to hormonal fluctuations leading to increased sebum secretion by sebaceous glands, abnormal keratinization of hair follicles and sebaceous glands, imbalance of skin microbiome, and inflammatory responses mediated by Cutibacterium acnes (*C. acnes*) [5]. The balance of the skin microbiome is crucial for maintaining skin health. In the pathogenesis of acne, disruption of the skin microbiome plays an important role [6]. Studies have shown that in the skin surface microbiota of acne patients, the over-proliferation of propionibacterium acnes may lead to a decrease in the number of other beneficial bacteria, and this imbalance of the microbiota may be closely related to the impairment of skin barrier function [2, 7]. This article reviews the action mechanisms of skin microorganisms such as *C. acnes* , *Malassezia*, Staphylococcus, and gut microorganisms in the formation of acne, and it clarifies the mutual relationship between the imbalance of skin microbiota and the formation of acne.  Table 1 provides a relatively detailed summary of the roles, mechanisms, and therapeutic implications of skin and gut microbiota in acne pathogenesis.

## 2. The Mechanism of Action of *C.* acnes in the Formation of Acne

*C.* acnes (Propionibacterium acnes, *P. acnes*, changed its name to Cutibacterium acnes, *C. acnes* [8]) is a gram-positive, nonspore-forming anaerobic bacillus of the phylum Actinomycetota. It is mainly rooted in the follicles of the sebaceous glands of human skin and is a normal skin-associated flora [9]. In early studies, Kelhala et al. [10] employed 16S rRNA gene sequencing technology to analyze the microbial composition of the pilosebaceous unit in acne patients and healthy individuals. They found that *C. acnes* dominated the microbial community structure in both groups, and its relative abundance exhibited a significant positive correlation with the clinical severity of acne. This observation led to the traditional view that the overproliferation of *C. acnes* is one of the core driving factors in acne pathogenesis. The pathogenic mechanisms of *C. acnes* involve multiple dimensions, including the regulation of abnormal sebum secretion, the mediation of chronic inflammatory responses, and the formation of biofilms to enhance colonization resistance.

However, with the advancement of technologies such as metagenomics and comparative genomics, this understanding has gradually been revised: the development of acne does not solely depend on the increased abundance of *C. acnes* [11, 12]. It is also dependent on the strain-specific pathogenic potential of this bacterium [13]. Metagenomic studies have confirmed that the phylotype diversity (rather than relative abundance) of the *C. acnes* community in acne lesions is significantly reduced—specifically, a decrease in both phylotype richness and evenness. A core characteristic of acne-associated phylotypes is the upregulated expression of virulence factors (VFs); these highly expressed VFs can exacerbate local inflammatory cascades by activating the host's innate and adaptive immune pathways [14]. Studies have demonstrated that the pathogenicity of *C. acnes* exhibits distinct strain dependence: certain phylotypes (e.g., phylotype IA1) are significantly associated with the development of inflammatory acne due to their stronger ability to activate the host's immune response [15], whereas other phylotypes (e.g., phylotype II) are more frequently distributed in healthy skin [16], suggesting that they may possess commensal or protective properties. This “quality over quantity” model, centered on strain pathogenicity, indicates that acne development is closely associated with differences in the structure and function of the microbial community.

In the future, targeted probiotic formulations, strain-specific vaccines, or precision antimicrobial strategies could be developed to achieve the selective elimination of highly pathogenic *C. acnes* strains, while preserving or even promoting the colonization and proliferation of beneficial commensal strains. By targeting the regulation of skin microbial balance to restore a healthy microbial community structure, this strategy can effectively avoid potential risks associated with broad-spectrum antimicrobial agents, such as skin microbial dysbiosis, bacterial drug resistance, and superinfection. It thus provides a new theoretical basis and technical direction for the clinical treatment of acne.

### 2.1. *C. acnes* Causes Overproduction of Sebum

Sebum is a complex lipid mixture secreted by sebaceous gland cells. Research has demonstrated that sebum serves as a metabolic substrate to promote the growth of *C. acnes*, while *C. acnes* stimulates sebaceous cells to upregulate the expression of cytokines, including interleukin-1*α* (IL-1*α*), interleukin-1β (IL-1β), and tumor necrosis factor-α (TNF-α) [17]. In addition, *C. acnes* enhances the activity of SZ95 sebaceous cells, leading to increased sebum secretion [18]. It also stimulates the epidermis to produce higher levels of corticotropin-releasing hormone (CRH), which subsequently activates the CRH receptor pathway to stimulate sebaceous glands [19]. Iinuma et al. [20] reported that *C. acnes* exacerbates androgen-related seborrhea by increasing the activity of diacylglycerol acyltransferase. Furthermore, through activation of the insulin-like growth factor-1 (IGF-1) receptor signaling pathway, *C. acnes* upregulates the expression of filaggrin, promotes keratinocyte proliferation and differentiation [21–23], thereby aggravating sebum hypersecretion and acne symptoms.

### 2.2. *C. acnes* Mediates the Inflammatory Response

The inflammatory response is integral to the pathogenesis of acne, currently classified as a “chronic inflammatory disease.” Inflammatory processes not only play a pivotal role in the development of acne but are also the underlying cause of its severe consequences, including acne scars, post-inflammatory hyperpigmentation, disfigurement, and significant psychological disorders [24]. *C. acnes* can activate both innate and adaptive immune responses, exacerbating inflammation through the release of inflammatory mediators.

**Figure 1:**
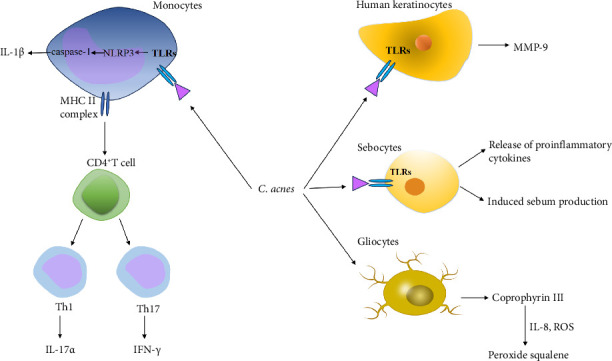
*C. acnes* plays multiple roles in acne development, including enhancing local inflammation and modulating immune responses. It stimulates sebocytes to release proinflammatory cytokines (IL-1α, IL-6, IL-8, IL-12, TNF-α, and IFN) through TLRs activation and activates the NLRP3 inflammasome and protease caspase-1, leading to the maturation and secretion of IL-1β. *C. acnes* also promotes CD4^+^ T cells to differentiate into Th17 and Th17/Th1 cells, promoting IL-17α and IFN-γ production. *C. acnes* increases the secretion of matrix metalloproteinase (MMP-9). *C. acnes* can also synthesize co-porphyrin III to act on gliocytes, promoting the release of catalysts IL-8 and ROS, thereby inducing the peroxidation of squalene.

In congenital immunity, *C. acnes* induces the production of cytokines such as IL-1*α*, IL-6, IL-8, and IL-12; tumor necrosis factor-α (TNF-α); and interferon (IFN) by stimulating toll-like receptors TLR-2 and TLR-4 on sebaceous cells (Figure 1) [25–27]. In addition, *C. acnes* activates the NLRP3 inflammasome and protease caspase-1, leading to the maturation and secretion of IL-1β (Figure 1) [21, 28]. In vitro studies by Jugeau et al. [29] demonstrated that *C. acnes* increases the expression of TLR-2 and TLR-4 in human keratinocytes and enhances the secretion of matrix metalloproteinase-9 (MMP-9), which contributes to inflammation (Figure 1). Furthermore, *C. acnes* mediates MMP expression by stimulating TNF-α production in human dermal fibroblasts (hDF) [30].

In adaptive immunity, Kistowska et al. [31] found that *C. acnes* induces T cell proliferation and differentiation, promoting CD4^+^ T cells to differentiate into Th17 and Th17/Th1 cells, thereby increasing the expression of proinflammatory cytokines IFN-γ and IL-17*α* and participating in the inflammatory response (Figure 1).

Other inflammatory mechanisms involve *C. acnes* synthesizing co-porphyrin III, which acts on glial cells to promote the release of IL-8, reactive oxygen species (ROS), and other catalysts, leading to squalene peroxidation and the formation of cytotoxic peroxide squalene (Figure 1) [32]. Studies have shown that *C. acnes* can produce a variety of invasive enzymes, such as esterase, which can not only damage the epithelial barrier but also hydrolyze the triglycerides in sebum into free fatty acids to stimulate the hair follicle and its surrounding tissues [33]. Hyaluronic acid lyase can degrade hyaluronic acid [34], which is an important component of the extracellular matrix and plays a role in the maintenance of skin structure and function.

### 2.3. *C. acnes* Forms a Dense Biofilm

Relevant studies have demonstrated that *C. acnes* forms dense biofilms and follows a complete path of biofilm formation, including adhesion, aggregation, maturation, and detachment [35]. Biofilms are complex microbial communities characterized by a three-dimensional network structure composed of extracellular polymeric substances (such as polysaccharides, proteins, and DNA) secreted by microorganisms [36]. These structures serve as protective barriers against host immune responses [37], effectively shielding the microbes from external threats. Jahns et al. [38] conducted controlled experiments and found that *C. acnes* isolated from acne patients more frequently formed biofilms within sebaceous glands of hair follicles. The formation of biofilms enhances the resistance of *C. acnes* to external stimuli, thereby conferring robust protection against the host's immune system. Burkhart et al. [39] proposed that biofilm formation increases the adhesion of *C. acnes* to the hair follicle wall, leading to enhanced keratinocyte cohesion and promoting the development of microcomedone. Consequently, the presence of biofilms facilitates the proliferation of *C. acnes*, contributing to recurrent and persistent inflammatory responses.

### 2.4. *C. acnes* Phylotypes Diversity Loss

*C. acnes* is currently classified into six major phylotypes: IA1, IA2, IB, IC, II, and III [40]. Based on single-locus sequence typing (SLST), these phylotypes can be further differentiated into more refined clonal complex subtypes, enabling high-resolution discrimination of strain-level differences. Recent studies indicate that loss of phylotypic diversity and disruption of community structure of this bacterium play a significant role in the pathogenesis of acne [41]. Metagenomic sequencing analyses have revealed significant differences in the phylotype composition of the skin microbiota between acne patients and healthy individuals, particularly reflected in the enrichment of high-virulence lineages [21]. Although *C. acnes* colonizes both acne-affected and healthy skin, acne-prone skin is frequently associated with the predominant proliferation of specific “acnegenic” phylotypes (such as IA1) and a reduction in overall phylotypic diversity. Multiple studies consistently demonstrate a positive correlation between acne severity and reduced strain diversity, a phenomenon observed not only on the faces of individuals with mild to moderate acne but also on the backs of those with severe acne [42]. Furthermore, the loss of phylotypic diversity can activate the innate immune system, thereby triggering inflammatory responses. Experimental evidence shows that, compared to combined stimulation with multiple phylotypes (IA1 + II + III), stimulation of skin explants with IA1 alone significantly upregulates the expression levels of innate immune markers such as IL-6, IL-8, IL-10, and IL-17 [43]. Case-control studies further support this conclusion, revealing that patients with severe acne often exhibit reduced phylotypic diversity along with dominant colonization by phylotype IA1 and SLST type A1 [40].

## 3. The Mechanism of Action of *Malassezia* in the Formation of Acne

*Malassezia,* a commensal lipophilic yeast residing on the human skin surface, has been confirmed to be explicitly associated with various skin diseases, including tinea versicolor, *Malassezia* folliculitis (MF), seborrheic dermatitis, psoriasis, and atopic dermatitis [44]. Among these associations, its correlation with acne has attracted extensive attention in recent years. Numata et al. [45] detected the *Malassezia* load on the skin of 100 acne patients and 28 healthy volunteers, and found that the number of *Malassezia* globosa on the skin of acne patients was significantly higher than that in the healthy control group, providing epidemiological evidence for the correlation between *Malassezia* and acne.

From the perspective of mechanisms, *Malassezia* can participate in the induction of acne through multiple pathways: First, it can hydrolyze triglycerides in sebum to produce irritating free fatty acids. These free fatty acids can directly stimulate hair follicles and surrounding tissues, induce polymorphonuclear neutrophil chemotaxis, trigger abnormal keratinization of hair follicle ducts, and promote keratinocytes to secrete proinflammatory cytokines [46–48]. Second, *Malassezia* can secrete various invasive enzymes such as esterases, phospholipases, proteases, and hyaluronidases. These enzymes not only disrupt the skin epithelial barrier, hair follicle walls, and surrounding tissues but also interfere with cell membrane signaling functions [49, 50], creating conditions for its further invasion of skin tissues. Third, molecular mechanism studies have shown that *Malassezia* can regulate the balance of cytokine secretion in keratinocytes via TLR-2 [51]. Furthermore, a study by Thomas et al. [52] confirmed that *Malassezia* can significantly upregulate the expression levels of proinflammatory cytokines (e.g., IL-1α, IL-6, IL-8, and TNF-α) in keratinocytes while downregulating the expression of the anti-inflammatory cytokine IL-10. The imbalance of these cytokines plays a crucial role in the morphological changes and apoptosis of keratinocytes. Currently, the specific mechanisms by which *Malassezia* contributes to the development and progression of acne require further in-depth investigation.

Notably, MF is an acneiform folliculitis with clinical manifestations similar to those of acne vulgaris. The two conditions are often confused in clinical practice and may coexist in the same patient. MF is an inflammatory skin disease caused by the overproliferation of *Malassezia* yeasts, with typical lesions presenting as monomorphic, pruritic papules or pustules and predilection sites including the hairline, face, and upper trunk. The overlapping clinical manifestations between MF and acne vulgaris pose challenges for their differential diagnosis [53].

However, there are fundamental differences in the etiology and pathogenesis between MF and acne vulgaris: the former is a fungal folliculitis, while the latter is mainly associated with bacterial colonization, excessive sebum secretion, and abnormal hair follicle keratinization. This difference directly affects the selection of treatment strategies—MF requires antifungal agents, whereas acne vulgaris often uses antibiotics, retinoids, and other medications. Failure to accurately differentiate the two conditions and subsequent incorrect treatment administration not only fails to control the disease but may also lead to delayed treatment or even exacerbation of fungal proliferation [54]. Therefore, improving the clinical ability to differentiate between MF and acne vulgaris is of great significance for formulating effective treatment regimens and improving patient prognosis.

## 4. The Mechanism of Action of Staphylococcus in the Formation of Acne

### 4.1. *Staphylococcus aureus* (*S. aureus*)

*S. aureus*, a gram-positive coccus, is a symbiotic bacterium of human skin and one of the main pathogens of human suppurative infections, which can cause a variety of community and hospital infections [55], including skin and soft tissue infections, pneumonia, endocarditis, and so on. There has been little discussion as to whether *S. aureus* is a causative factor in acne. Through 16S rRNA high-throughput sequencing, Dreno et al. [56] found that in acne patients, *S. aureus* has a higher content on the surface of acne, papules, and pustules, and the amount is positively correlated with the severity of acne, compared with the skin without lesions. Whether *S. aureus* plays a role in the pathogenesis of acne is still controversial.

### 4.2. *Staphylococcus epidermidis* (*S. epidermidis*)

*S. epidermidis* is a coagulase-negative, gram-positive coccus that forms part of the normal human skin and mucosal flora, predominantly residing in the armpits, head, and nostrils [57, 58]. Characterized by its ability to evade innate immune defenses and exhibit low virulence, *S. epidermidis* is an important opportunistic pathogen and a significant contributor to nosocomial infections, particularly through biofilm formation on indwelling medical devices, leading to persistent infections [57, 59]. However, *S. epidermidis* also plays a crucial role as a component of the healthy skin microbiome, acting as a microbial barrier and contributing to skin homeostasis. Phenol-soluble modulins produced by *S. epidermidis* selectively inhibit pathogens like *S. aureus* and Streptococcus pyogenes, and can synergize with host antimicrobial peptides (AMPs) to enhance their bactericidal effects [60]. In addition, *S. epidermidis* helps maintain inflammatory homeostasis by inhibiting excessive cytokine release following minor epidermal injuries [61]. Thomsen et al. [62] reported that *S. epidermidis* accounted for 6.8% to 47.3% of microorganisms colonizing the follicular sebaceous gland units in acne patients, second only to *C. acnes*. Although there is no definitive evidence that *S. epidermidis* actively contributes to acne development, Wang et al. [63] demonstrated that *S. epidermidis* could inhibit *C. acnes* growth via succinic acid produced during fermentation. Furthermore, *S. epidermidis* secretes staphylococcal lipoteichoic acid, which mitigates *C. acnes*–induced inflammation by upregulating miR-143 expression and downregulating TLR-2 expression in keratinocytes [48]. Thus, *S. epidermidis* exerts beneficial effects in acne by inhibiting *C. acnes* proliferation, suggesting that acne onset may be associated with an imbalance between these two bacterial species.

### 4.3. The Complex Role of the Staphylococcus in Acne

In summary, the role of Staphylococcus spp. in acne pathogenesis is characterized by considerable complexity and functional duality. Although enrichment of *S. aureus* has been observed in acne lesions, a causal relationship has not been established; its involvement remains correlative and may reflect opportunistic colonization rather than primary pathogenesis. In contrast, *S. epidermidis* exemplifies the dualistic nature of cutaneous commensals: it serves as a key protector of skin homeostasis through multiple mechanisms that potently inhibit *C. acnes*, yet under specific conditions—such as in the context of medical device–associated biofilm formation—it can act as an opportunistic pathogen.

Therefore, in clinical practice, this understanding implies that broad-spectrum anti-staphylococcal treatments (such as topical clindamycin targeting all staphylococci) are not warranted. Instead, employing strategies that enhance the protective function of *S. epidermidis* may represent a more effective approach to acne management.

## 5. The Effect of Skin Microbiota Imbalance on Acne

Skin microbiota and human skin cells collectively form a complex ecological environment, constituting an integral part of the skin defense system (skin biological barrier), which is one of the primary functions of the skin barrier. The homeostasis of the skin microbiome represents a dynamic equilibrium between the microbial community on the skin surface and the host. This balance is crucial for maintaining healthy skin. Disruption of this balance, either between microorganisms or between microorganisms and the host, can lead to the proliferation of pathogenic microorganisms or overgrowth of normal flora, abnormal immune responses, and alterations in skin barrier function, potentially causing or exacerbating various skin diseases. The development of acne is closely associated with such imbalances in the skin microbiome.

On the one hand, the normal skin microbiota plays a vital role in maintaining the skin's physiological functions. For instance, *S. epidermidis* can limit the excessive proliferation of *C. acnes* and inhibit the inflammatory response induced by *C. acnes*. On the other hand, *C. acnes* maintains a low pH in the sebaceous unit of hair follicles through the hydrolysis of triacylglycerol and secretion of propionic acid, thereby restricting the growth of *S. aureus* and Streptococcus pyogenes [63]. *S. epidermidis* also inhibits the formation of pathogenic S. aureus biofilm by secreting bacteriocin [57]. Akaza et al. [64] found that the dominant strains in the contents of inflammatory acne lesions in acne patients were *C. acnes* and *Malassezia*. Kwon et al. [65] observed that compared to healthy individuals, there was a higher prevalence of gastrointestinal streptococci in the hair follicles of acne patients, with the quantity positively correlated with the severity of acne. Multiple studies have shown that acne is a skin disease caused by an imbalance of the skin microbiome, and the balance of the skin microbiome is essential for the homeostasis of the skin. The pursuit of treatments that restore the balance of the skin microecology not only helps to restore the damaged skin barrier function but also reduces the risk of recurrent acne, which has great potential in future acne treatment.

## 6. Gut–Skin Axis

The concept of “intestinal-skin comorbidity” in traditional Chinese medicine posits a close pathological relationship between the occurrence of skin diseases and intestinal dysfunction. The “gut–skin axis” refers to the ecosystem of microbial interactions between the gut and the skin. Both the organs can communicate through immune, endocrine, circulatory, and neural pathways [66]. Developmental biology studies have shown that skin cells and intestinal cells are derived from the same embryonic layer and have similar signal transduction mechanisms. At the same time, skin and intestine are also key immune and neuroendocrine organs, and there is a close correlation between intestinal health and skin homeostasis, and a relatively mature “gut–skin axis” theory has been formed at present [67].

Numerous studies have demonstrated intricate, immune, metabolic, and neuroendocrine connections between the intestinal microbiota and skin microbiota [68]. In terms of immune interactions, the gut flora plays a crucial role in maintaining cutaneous immune homeostasis by modulating both innate and adaptive immunity [69]. Specifically, gut flora metabolizes dietary fiber into short-chain fatty acids (SCFAs), which induce regulatory T cells (Tregs) to downregulate inflammatory mediators and participate in immune regulation [70]. Regarding metabolic associations, gut flora and its metabolites can alter intestinal mucosal permeability, thereby entering the circulatory system and influencing the skin barrier function. Disruption of gut flora may impair intestinal barrier function, permitting harmful substances (such as endotoxins) and bacteria to enter the bloodstream, thus affecting skin homeostasis. Inflammatory skin conditions are often associated with disturbances in both skin and gut microbiota [71, 72]. Thompson et al. [73] reported significant alterations in both skin and gut microbiota in acne patients. Disruptions in gut flora can increase intestinal mucosal epithelial permeability, activate effector T cells, and disrupt the balance between these cells and Tregs [74, 75]. In addition, disruptions in gut flora can lead to the release of inflammatory mediators, such as metabolites from gut microbiota, into the circulatory system, which can control cell proliferation via the mTOR signaling pathway [76], thereby exacerbating systemic inflammatory responses and inducing acne. Known as the “second brain,” the gut has neuroendocrine connections where gut flora can synthesize and release neurotransmitters such as norepinephrine, serotonin, and acetylcholine through the “gut–brain axis.” This process stimulates nerve pathways, triggers the release of hormones from intestinal endocrine cells, and subsequently produces widespread systemic effects, including inflammation [77].

In recent years, research on gut microbiota has provided novel insights into the pathogenesis and therapeutic strategies for acne. The “gut–skin axis” has become a focus of investigation in this field, revealing a complex bidirectional regulatory relationship between the gut and the skin. Although accumulating evidence indicates that certain skin disorders are closely associated with gut dysbiosis and impaired intestinal barrier function, the precise molecular mechanisms through which gut microbiota influence skin condition remain incompletely elucidated. Beyond immune regulation, dietary components and microbial metabolites—such as SCFAs and bile acids—can modulate intestinal mucosal permeability and physiological status, facilitating the systemic circulation of various bioactive substances that ultimately affect the skin microenvironment [75].

Based on the gut–skin axis theory, probiotics and prebiotics, as effective approaches to modulating gut microbial balance, exhibit considerable potential for improving skin health. Concurrently, the integration of well-characterized prebiotics or probiotic metabolites into daily skincare products demonstrates promising clinical translational value. For instance, several gut microbiota–derived metabolites have been incorporated into cosmetic formulations in attempts to enhance skin barrier function or regulate local inflammation. Further elucidating the role of gut microorganisms in skin barrier homeostasis and developing individualized skin health management strategies based on these mechanisms represent a highly promising direction for future clinical applications [78]. In addition, increased dietary fiber intake, the development of drugs targeting gut microbiota, fecal microbiota transplantation (FMT), and combined intervention strategies offer new perspectives for improving gut and skin health, with considerable potential for future acne treatment paradigms.

Although targeting the “gut–skin axis” holds tremendous prospects for acne treatment, the current level of evidence supporting its clinical application varies substantially. Mechanistic and preclinical studies have provided a solid theoretical foundation, demonstrating that gut microbiota can influence skin status by modulating systemic immune and inflammatory responses, thereby validating the existence of the “gut–skin axis” [79]. Preliminary clinical observations have also identified associations between gut dysbiosis and acne occurrence [80], and several small-scale studies have reported that probiotic interventions can ameliorate skin symptoms [81, 82]. However, there remains a lack of high-quality clinical evidence from large-scale randomized controlled trials (RCTs)—such studies are needed to definitively establish the efficacy, safety, and optimal application parameters of interventions such as probiotics, prebiotics, or FMT in acne treatment [83]. Therefore, although gut microbiota modulation represents a highly promising research direction, it is currently recommended as an adjunctive strategy rather than a first-line treatment for acne until more robust clinical evidence becomes available.

## 7. Summary and Future Prospects

Acne is a common inflammatory skin disorder that not only causes significant physical discomfort—such as pain, pruritus, and visible lesions—but also exerts profound negative effects on psychological well-being, including anxiety, depression, and social impairment. In microbiome research related to acne, *C. acnes* has long been central to investigations. Nevertheless, the roles of other bacteria and fungi in acne pathogenesis and progression remain inadequately explored, warranting further investigation into their species diversity, functional properties, and interaction mechanisms with the host. Furthermore, the “gut–skin axis” theory provides a novel perspective for systemically understanding acne etiology and treatment, highlighting the interactive mechanisms between gut and skin microbiomes.

Current research on acne treatment is advancing toward diversified and innovative strategies [84], including acne vaccines, novel microneedle patch-based drug delivery systems, genetically engineered *C. acnes*, modified painless photodynamic therapy (M-PDT), ultra-pulsed CO_2_ fractional lasers, and integrated traditional Chinese and Western medicine approaches. These represent promising future therapeutic directions.

Several core scientific questions remain in acne microbiome research, outlining key areas for future investigation:

First, the strain-specific pathogenic mechanisms of *C. acnes* are not yet fully elucidated. Systematic exploration is needed to decipher the molecular-level functional differences between proinflammatory (e.g., phylotype IA1) and commensal (e.g., phylotype II) strains, including their metabolic profiles and interactions with keratinocytes. In addition, identifying key virulence determinants and developing treatment strategies that precisely target pathogenic strains while preserving beneficial ones are crucial.

Second, microbiome-targeted therapies still lack high-level clinical evidence. Interventions such as probiotics, phage therapy, microbial transplantation, prebiotics, and acne vaccines require validation through large-scale RCTs to assess long-term efficacy and safety. Standardized production and personalized application protocols for these treatments also represent major challenges for clinical translation.

Third, the causal mechanisms underlying the “gut–skin axis” remain incompletely understood. Current evidence is largely correlative; further research is essential to elucidate how gut microbial metabolites modulate skin microbiota composition and follicular inflammation via systemic circulation. Defining the specific molecular pathways and causal relationships in acne development is imperative.

Fourth, integrated strategies combining microbial modulation with conventional therapies require systematic evaluation. Future studies should examine whether microbiome-based interventions synergize with conventional treatments to enhance efficacy, reduce antibiotic resistance, and minimize adverse effects. Furthermore, optimized combinatorial regimens must be established to achieve effective and sustained acne prevention.

## Figures and Tables

**Table 1:** The role and mechanisms of skin and gut microbiota in acne pathogenesis and treatment implications.

Microorganism/aspect	Key associations and characteristics	Mechanisms in acne pathogenesis	Clinical and therapeutic implications
Cutibacterium acnes (*C. acnes)*	① Dominant colonizer of pilosebaceous units. ② Phylotype diversity loss (e.g., enrichment of IA1, reduction of II) correlates with severity. ③ “Quality over quantity”: Strain-specific pathogenicity is crucial.	① Sebum production: Activates IGF-1R, upregulates filaggrin, stimulates CRH pathways, and increases DGAT activity. ② Inflammation: Activates TLR-2/4, NLRP3 inflammasome (IL-1β), promotes Th17/Th1 responses (IFN-γ, IL-17), and induces proinflammatory cytokines (IL-6, IL-8, TNF-α). ③ Biofilm formation: Enhances antibiotic resistance, keratinocyte cohesion, and microcomedone formation. ④ Phylotypes diversity loss: In acne lesions, *C. acnes* loses phylotype diversity with high-virulence IA1 enrichment, and its stimulation of skin explants upregulates IL-6, IL-8, and other inflammatory markers.	① Future therapies should target pathogenic strains (e.g., IA1) while preserving beneficial ones (e.g., II). ② Precision antimicrobials, probiotics, and vaccines are under investigation. ③ Broad-spectrum antibiotics may disrupt commensal balance.

Malassezia spp.	① Lipophilic yeast; higher abundance of *Malassezia globosa* in acne patients. ② Causes *Malassezia* folliculitis (MF), often misdiagnosed as acne vulgaris.	① Free fatty acids: Hydrolyzes triglycerides ⟶ releases irritating FFAs ⟶ induces inflammation and hyperkeratosis. ② Enzymes: Secretes esterases, phospholipases, proteases, and hyaluronidases ⟶ disrupts epithelial barrier and follicular integrity. ③ Immunomodulation: Upregulates proinflammatory cytokines (IL-1*α*, IL-6, IL-8, and TNF-α) and downregulates IL-10 via TLR-2.	① Crucial to differentiate MF from acne vulgaris for correct treatment. ② Requires antifungal therapy, not antibiotics. ③ Potential for topical prebiotics/probiotics to restore balance.

Staphylococcus spp.	① *S. aureus*: Enriched in lesions; correlation with severity but causal role unproven. ② *S. epidermidis*: Commensal with dual role; inhibits *C. acnes* via succinic acid and lipoteichoic acid (downregulates TLR-2).	① *S. aureus*: Potential opportunistic pathogen in disrupted skin. ② *S. epidermidis*: Antagonizes *C. acnes* and modulates inflammation (e.g., via miR-143). Maintains skin homeostasis when balanced.	① *S. epidermidis* is a protective commensal; strategies to enhance its activity are promising. ② Broad-spectrum anti-staphylococcal agents (e.g., clindamycin) may be counterproductive. ③ Topical probiotics containing *S. epidermidis* are a potential therapeutic avenue.

Skin microbiome dysbiosis	① Acne is associated with a loss of overall microbial diversity and balance. ② Disruption of the competitive equilibrium between *C. acnes*, *S. epidermidis*, and other commensals.	① Reduction in beneficial microbes impairs the skin's biological barrier function. ② Overgrowth of pathogenic phylotypes (e.g., *C. acnes* IA1) triggers immune responses.	① Therapeutic goal is to restore ecological balance rather than eradicate bacteria. ② Prebiotics, postbiotics, and microbial transplantation are emerging strategies.

Gut–skin axis	① Gut dysbiosis and increased intestinal permeability are observed in acne patients. ② Connects via immune, metabolic, and neuroendocrine pathways.	① Immune: Gut microbiota modulates systemic immunity (e.g., SCFAs ⟶ Tregs). ② Metabolic: Bacterial metabolites (SCFAs, bile acids) enter circulation and affect skin inflammation and barrier function. ③ Neuroendocrine: Gut microbes influence stress responses and inflammation via the gut–brain–skin axis.	① Probiotics, prebiotics, and dietary modifications (e.g., high fiber) show potential to ameliorate acne. ② Fecal microbiota transplantation (FMT) is being explored. ③ Currently an adjunctive strategy; more RCTs are needed to confirm efficacy and protocols.

## Data Availability

Data sharing is not applicable to this article as no datasets were generated or analyzed during the current study.

## References

[1] Chen Y. E., Fischbach M. A., Belkaid Y. (2018). Skin Microbiota-Host Interactions. *Nature*.

[2] Harris-Tryon T. A., Grice E. A. (2022). Microbiota and Maintenance of Skin Barrier Function. *Science*.

[3] Zhang X., Zheng P., Ye S.-Z. (2024). Microbiome: Role in Inflammatory Skin Diseases. *Journal of Inflammation Research*.

[4] Li Y., Hu X., Dong G., Wang X., Liu T. (2024). Acne Treatment: Research Progress and New Perspectives. *Frontiers of Medicine*.

[5] Kim J., Park T., Kim H.-J., An S., Sul W. J. (2021). Inferences in Microbial Structural Signatures of Acne Microbiome and Mycobiome. *Journal of Microbiology*.

[6] Zhu Y., Yu X., Cheng G. (2023). Human Skin Bacterial Microbiota Homeostasis: A Delicate Balance Between Health and Disease. *mLife*.

[7] Zhu H., Lou F., Yin Q. (2017). RIG-I Antiviral Signaling Drives interleukin-23 Production and Psoriasis-Like Skin Disease. *EMBO Molecular Medicine*.

[8] Dréno B., Pécastaings S., Corvec S., Veraldi S., Khammari A., Roques C. (2018). Cutibacterium acnes (Propionibacterium acnes) and Acne Vulgaris: A Brief Look at the Latest Updates. *Journal of the European Academy of Dermatology and Venereology*.

[9] Ahle C. M., Feidenhansl C., Brüggemann H. (2023). Cutibacterium acnes. *Trends in Microbiology*.

[10] Kelhälä H., Aho V. T. E., Fyhrquist N. (2018). Isotretinoin and Lymecycline Treatments Modify the Skin Microbiota in Acne. *Experimental Dermatology*.

[11] Dessinioti C., Katsambas A. (2017). Propionibacterium acnes and Antimicrobial Resistance in Acne. *Clinics in Dermatology*.

[12] Omer H., McDowell A., Alexeyev O. A. (2017). Understanding the Role of Propionibacterium acnes in Acne Vulgaris: the Critical Importance of Skin Sampling Methodologies. *Clinics in Dermatology*.

[13] O’Neill A. M., Gallo R. L. (2018). Host-Microbiome Interactions and Recent Progress into Understanding the Biology of Acne Vulgaris. *Microbiome*.

[14] Yu T., Xu X., Liu Y. (2024). Multi-Omics Signatures Reveal Genomic and Functional Heterogeneity of Cutibacterium acnes in Normal and Diseased Skin. *Cell Host & Microbe*.

[15] Cheung C. T., Lancien U., Corvec S. (2024). Pro‐Inflammatory Activity of *Cutibacterium acnes* Phylotype_*IA1*_ and Extracellular Vesicles: An in vitro stuVy. *Experimental Dermatology*.

[16] Spittaels K.-J., Ongena R., Zouboulis C. C., Crabbé A., Coenye T. (2020). Cutibacterium acnes Phylotype I and II Strains Interact Differently With Human Skin Cells. *Frontiers in Cellular and Infection Microbiology*.

[17] Vowels B. R., Yang S., Leyden J. J. (1995). Induction of proinflammatory cytokines by a soluble factor of Propionibacterium acnes: implications for chronic inflammatory acne. *Infection and Immunity*.

[18] Huang Yu-C., Yang C.-H., Li T.-T., Zouboulis C. C., Hsu H.-C. (2015). Cell-free extracts of Propionibacterium acnes stimulate cytokine production through activation of p38 MAPK and Toll-like receptor in SZ95 sebocytes. *Life Sciences*.

[19] Zouboulis C. C. (2009). Propionibacterium acnes and sebaceous lipogenesis: a love-hate relationship?. *Journal of Investigative Dermatology*.

[20] Iinuma K., Sato T., Akimoto N. (2009). Involvement of Propionibacterium acnes in the augmentation of lipogenesis in hamster sebaceous glands in vivo and in vitro. *Journal of Investigative Dermatology*.

[21] Lee Y. B., Byun E. J., Kim H. S. (2019). Potential Role of the Microbiome in Acne: A Comprehensive Review. *Journal of Clinical Medicine*.

[22] Li Z. J., Choi D. K., Sohn K. C. (2014). Propionibacterium acnes activates the NLRP3 inflammasome in human sebocytes. *Journal of Investigative Dermatology*.

[23] Rahaman S. M. A., De D., Handa S. (2016). Association of insulin-like growth factor (IGF)-1 gene polymorphisms with plasma levels of IGF-1 and acne severity. *Journal of the American Academy of Dermatology*.

[24] Williams H. C., Dellavalle R. P., Garner S. (2012). Acne vulgaris. *The Lancet*.

[25] Nakatsuji T., Liu Yu-T., Huang C., Gallo R. L., Huang C. M., Huang C.-M. (2008). Vaccination targeting a surface sialidase of P. acnes: implication for new treatment of acne vulgaris. *PLoS One*.

[26] Kistowska M., Gehrke S., Jankovic D. (2014). IL-1β drives inflammatory responses to propionibacterium acnes in vitro and in vivo. *Journal of Investigative Dermatology*.

[27] Savitri D., Wahyuni S., Bukhari A. (2023). Anti-inflammatory effects of banana (Musa balbisiana) peel extract on acne vulgaris: In vivo and in silico study. *Journal of Taibah University Medical Sciences*.

[28] Qin M., Pirouz A., Kim M.-H., Krutzik S. R., Garbán H. J., Kim J. (2014). Propionibacterium acnes Induces IL-1β secretion via the NLRP3 inflammasome in human monocytes. *Journal of Investigative Dermatology*.

[29] Jugeau S., Tenaud I., Knol A. C. (2005). Induction of toll-like receptors by Propionibacterium acnes. *British Journal of Dermatology*.

[30] Choi J.-Y., Piao M. S., Lee J.-B., Oh J. S., Lee S. C. (2008). Propionibacterium acnes stimulates pro-matrix metalloproteinase-2 expression through tumor necrosis factor-alpha in human dermal fibroblasts. *Journal of Investigative Dermatology*.

[31] Kistowska M., Meier B., Proust T. (2015). Propionibacterium acnes promotes Th17 and Th17/Th1 responses in acne patients. *Journal of Investigative Dermatology*.

[32] Bowe W. P., Patel N., Logan A. C. (2012). Acne vulgaris: the role of oxidative stress and the potential therapeutic value of local and systemic antioxidants. *Journal of Drugs in Dermatology*.

[33] Nakatsuji T., Kao M. C., Zhang L., Zouboulis C. C., Gallo R. L., Huang C.-M. (2010). Sebum free fatty acids enhance the innate immune defense of human sebocytes by upregulating beta-defensin-2 expression. *Journal of Investigative Dermatology*.

[34] Nazipi S., Stødkilde K., Scavenius C., Brüggemann H. (2017). The Skin Bacterium Propionibacterium acnes Employs Two Variants of Hyaluronate Lyase with Distinct Properties. *Microorganisms*.

[35] Kuehnast T., Cakar F., Weinhäupl T. (2018). Comparative analyses of biofilm formation among different Cutibacterium acnes isolates. *Int J Med Microbiol*.

[36] Coenye T., Spittaels K.-J., Achermann Y. (2022). The role of biofilm formation in the pathogenesis and antimicrobial susceptibility of Cutibacterium acnes. *Biofilms*.

[37] Loss M., Thompson K. G., Agostinho-Hunt A. (2021). Noninflammatory comedones have greater diversity in microbiome and are more prone to biofilm formation than inflammatory lesions of acne vulgaris. *International Journal of Dermatology*.

[38] Jahns A. C., Lundskog B., Ganceviciene R. (2012). An increased incidence of Propionibacterium acnes biofilms in acne vulgaris: a case-control study. *British Journal of Dermatology*.

[39] Burkhart C. G., Burkhart C. N. (2007). Expanding the microcomedone theory and acne therapeutics: Propionibacterium acnes biofilm produces biological glue that holds corneocytes together to form plug. *Journal of the American Academy of Dermatology*.

[40] Paugam C., Corvec S., Saint‐Jean M. (2017). Propionibacterium acnes phylotypes and acne severity: an observational prospective study. *Journal of the European Academy of Dermatology and Venereology*.

[41] Fitz-Gibbon S., Tomida S., Chiu B.-H. (2013). Propionibacterium acnes strain populations in the human skin microbiome associated with acne. *Journal of Investigative Dermatology*.

[42] Dagnelie M.-A., Montassier E., Khammari A., Mounier C., Corvec S., Dréno B. (2019). Inflammatory skin is associated with changes in the skin microbiota composition on the back of severe acne patients. *Experimental Dermatology*.

[43] Dagnelie M.-A., Corvec S., Saint-Jean M., Nguyen J.-M., Khammari A., Dréno B. (2019). Cutibacterium acnes phylotypes diversity loss: a trigger for skin inflammatory process. *Journal of the European Academy of Dermatology and Venereology*.

[44] Gaitanis G., Magiatis P., Hantschke M., Bassukas I. D., Velegraki A. (2012). The Malassezia genus in skin and systemic diseases. *Clinical Microbiology Reviews*.

[45] Numata S., Akamatsu H., Akaza N., Yagami A., Nakata S., Matsunaga K. (2014). Analysis of facial skin-resident microbiota in Japanese acne patients. *Dermatology*.

[46] Naglik J., Rodgers C. A., Shirlaw P. (2003). Differential expression of Candida albicans secreted aspartyl proteinase and phospholipase B genes in humans correlates with active oral and vaginal infections. *The Journal of Infectious Diseases*.

[47] Vijaya Chandra S. H., Srinivas R., Dawson T. L., Common J. E. (2020). Cutaneous Malassezia: Commensal, Pathogen, or Protector?. *Frontiers in Cellular and Infection Microbiology*.

[48] Xu H., Li H. (2019). Acne, the Skin Microbiome, and Antibiotic Treatment. *American Journal of Clinical Dermatology*.

[49] D Coutinho S., Paula C. R. (2000). Proteinase, phospholipase, hyaluronidase and chondroitin-sulphatase production by Malassezia pachydermatis. *Medical Mycology*.

[50] Riciputo R. M., Oliveri S., Micali G., Sapuppo A. (1996). Phospholipase activity in Malassezia furfur pathogenic strains. *Mycoses*.

[51] Baroni A., Orlando M., Donnarumma G. (2006). Toll-like receptor 2 (TLR2) mediates intracellular signalling in human keratinocytes in response to Malassezia furfur. *Archives of Dermatological Research*.

[52] Thomas D. S., Ingham E., Bojar R. A., Holland K. T. (2008). In vitro modulation of human keratinocyte pro- and anti-inflammatory cytokine production by the capsule of Malassezia species. *FEMS Immunology and Medical Microbiology*.

[53] Paichitrojjana A., Chalermchai T. (2022). The Prevalence, Associated Factors, and Clinical Characterization of Malassezia folliculitis in Patients Clinically Diagnosed with Acne Vulgaris. *Clinical, Cosmetic and Investigational Dermatology*.

[54] Del Rosso J. Q., Silverberg N., Zeichner J. A. (2016). When Acne is Not Acne. *Dermatologic Clinics*.

[55] Chessa D., Ganau G., Mazzarello V. (2015). An overview of Staphylococcus epidermidis and Staphylococcus aureus with a focus on developing countries. *J Infect Dev Ctries*.

[56] Dreno B., Martin R., Moyal D., Henley J. B., Khammari A., Seité S. (2017). Skin microbiome and acne vulgaris: Staphylococcus, a new actor in acne. *Experimental Dermatology*.

[57] Christensen G. J. M., Brüggemann H. (2014). Bacterial skin commensals and their role as host guardians. *Beneficial Microbes*.

[58] Otto M. (2009). Staphylococcus epidermidis--the ‘accidental’ pathogen. *Nature Reviews Microbiology*.

[59] Costerton J. W., Stewart P. S., Greenberg E. P. (1999). Bacterial biofilms: a common cause of persistent infections. *Science*.

[60] Grice E. A., Segre J. A. (2011). The skin microbiome. *Nature Reviews Microbiology*.

[61] Lai Y., Di Nardo A., Nakatsuji T. (2009). Commensal bacteria regulate Toll-like receptor 3-dependent inflammation after skin injury. *Nature Medicine*.

[62] Bek-Thomsen M., Lomholt H. B., Kilian M. (2008). Acne is not associated with yet-uncultured bacteria. *Journal of Clinical Microbiology*.

[63] Wang Y., Kuo S., Shu M. (2014). Staphylococcus epidermidis in the human skin microbiome mediates fermentation to inhibit the growth of Propionibacterium acnes: implications of probiotics in acne vulgaris. *Applied Microbiology and Biotechnology*.

[64] Akaza N., Takasaki K., Nishiyama E. (2022). The Microbiome in Comedonal Contents of Inflammatory Acne Vulgaris is Composed of an Overgrowth of Cutibacterium Spp. and Other Cutaneous Microorganisms. *Clinical, Cosmetic and Investigational Dermatology*.

[65] Kwon H. H., Yoon J. Y., Park S. Y., Suh D. H. (2013). Analysis of distribution patterns of Propionibacterium acnes phylotypes and Peptostreptococcus species from acne lesions. *British Journal of Dermatology*.

[66] Arck P., Handjiski B., Hagen E. (2010). Is there a gut-brain-skin axis. *Experimental Dermatology*.

[67] O’Neill C. A., Monteleone G., McLaughlin J. T., Paus R. (2016). The gut-skin axis in health and disease: A paradigm with therapeutic implications. *BioEssays*.

[68] Park D. H., Kim J. W., Park Hi-J., Hahm D.-H. (2021). Comparative Analysis of the Microbiome across the Gut-Skin Axis in Atopic Dermatitis. *International Journal of Molecular Sciences*.

[69] Catinean A., Neag M. A., Mitre A. O., Bocsan C. I., Buzoianu A. D. (2019). Microbiota and Immune-Mediated Skin Diseases-An Overview. *Microorganisms*.

[70] Xiao X., Hu X., Yao J. (2023). The role of short-chain fatty acids in inflammatory skin diseases. *Frontiers in Microbiology*.

[71] Carucci L., Nocerino R., Paparo L. (2022). Therapeutic effects elicited by the probiotic Lacticaseibacillus rhamnosus GG in children with atopic dermatitis. The results of the ProPAD trial. *Pediatric Allergy & Immunology*.

[72] Chen H.-L., Zeng Yi-B., Zhang Z.-Y. (2021). Gut and Cutaneous Microbiome Featuring Abundance of Lactobacillus reuteri Protected Against Psoriasis-Like Inflammation in Mice. *Journal of Inflammation Research*.

[73] Thompson K. G., Rainer B. M., Antonescu C. (2020). Minocycline and Its Impact on Microbial Dysbiosis in the Skin and Gastrointestinal Tract of Acne Patients. *Annals of Dermatology*.

[74] Brown E. M., Kenny D. J., Xavier R. J. (2019). Gut Microbiota Regulation of T Cells During Inflammation and Autoimmunity. *Annual Review of Immunology*.

[75] Mahmud M. R., Akter S., Tamanna S. K. (2022). Impact of gut microbiome on skin health: gut-skin axis observed through the lenses of therapeutics and skin diseases. *Gut Microbes*.

[76] Noureldein M. H., Eid A. A. (2018). Gut microbiota and mTOR signaling: Insight on a new pathophysiological interaction. *Microbial Pathogenesis*.

[77] Pondeljak N., Lugović-Mihić L. (2020). Stress-induced Interaction of Skin Immune Cells, Hormones, and Neurotransmitters. *Clinical Therapeutics*.

[78] Lin Xu, Yu Z., Liu Y. (2025). Gut-X axis. *Imeta*.

[79] Bowe W. P., Logan A. C. (2011). Acne vulgaris, probiotics and the gut-brain-skin axis-back to the future?. *Gut Pathogens*.

[80] Deng Y., Wang H., Zhou J., Mou Y., Wang G., Xiong X. (2018). Patients with Acne Vulgaris Have a Distinct Gut Microbiota in Comparison with Healthy Controls. *Acta Dermato-Venereologica*.

[81] Jung G. W., Tse J. E., Guiha I., Rao J. (2013). Prospective, randomized, open-label trial comparing the safety, efficacy, and tolerability of an acne treatment regimen with and without a probiotic supplement and minocycline in subjects with mild to moderate acne. *Journal of Cutaneous Medicine and Surgery*.

[82] Fang Z., Pan T., Li L. (2022). Bifidobacterium longum mediated tryptophan metabolism to improve atopic dermatitis via the gut-skin axis. *Gut Microbes*.

[83] Boby A., Lee G., Natarelli N., Correa L. (2024). Using probiotics to treat acne vulgaris: systematic review. *Archives of Dermatological Research*.

[84] Layton A. M., Ravenscroft J. (2023). Adolescent acne vulgaris: current and emerging treatments. *The Lancet Child & Adolescent Health*.

